# Breast cancer risk assessment using genetic variants and risk factors in a Singapore Chinese population

**DOI:** 10.1186/bcr3678

**Published:** 2014-06-18

**Authors:** Charmaine Pei Ling Lee, Astrid Irwanto, Agus Salim, Jian-min Yuan, Jianjun Liu, Woon Puay Koh, Mikael Hartman

**Affiliations:** 1 NUS Graduate School for Integrative Sciences and Engineering, National University of Singapore, 21 Lower Kent Ridge Road, Singapore 119077, Singapore; 2 Saw Swee Hock School of Public Health, National University of Singapore, 21 Lower Kent Ridge Road, Singapore 119077, Singapore; 3 Human Genetics, Genome Institute of Singapore, 60 Biopolis Street, Singapore 138672, Singapore; 4 Department of Mathematics and Statistics, La Trobe University, Kingsbury Drive, Bundoora, VIC 3086, Australia; 5 Division of Cancer Control and Population Sciences, University of Pittsburgh Cancer Institute; and Department of Epidemiology, Graduate School of Public Health, University of Pittsburgh, 4200 Fifth Avenue, Pittsburgh, PA 15260, USA; 6 Duke-NUS Graduate Medical School Singapore, 2 Jalan Bukit Merah, Singapore 169547, Singapore; 7 Department of Surgery, National University Hospital, 5 Lower Kent Ridge Road, Singapore 119074, Singapore; 8 Department of Medical Epidemiology and Biostatistics, Karolinska Institutet, Nil, Stockholm SE-171 77, Sweden

## Abstract

**Introduction:**

Genetic variants for breast cancer risk identified in genome-wide association studies (GWAS) in Western populations require further testing in Asian populations. A risk assessment model incorporating both validated genetic variants and established risk factors may improve its performance in risk prediction of Asian women.

**Methods:**

A nested case-control study of female breast cancer (411 cases and 1,212 controls) within the Singapore Chinese Health Study was conducted to investigate the effects of 51 genetic variants identified in previous GWAS on breast cancer risk. The independent effect of these genetic variants was assessed by creating a summed genetic risk score (GRS) after adjustment for body mass index and the Gail model risk factors for breast cancer.

**Results:**

The GRS was an independent predictor of breast cancer risk in Chinese women. The multivariate-adjusted odds ratios (95% confidence intervals) of breast cancer for the second, third, and fourth quartiles of the GRS were 1.26 (0.90 to 1.76), 1.47 (1.06 to 2.04) and 1.75 (1.27 to 2.41) respectively (*P* for trend <0.001). In addition to established risk factors, the GRS improved the classification of 6.2% of women for their absolute risk of breast cancer in the next five years.

**Conclusions:**

Genetic variants on top of conventional risk factors can improve the risk prediction of breast cancer in Chinese women.

## Introduction

Breast cancer is a heterogeneous disease that is associated with genetic and environmental factors. Prior to genetic studies, investigations have mainly revolved around the presence of a family history, hormonal and reproductive-related risk factors [1-3] with the effects of other lifestyle factors being queried recently. To date, the risk of disease has been shown to increase with a woman’s age, age at menopause, age at first live birth, previous occurrence of atypical hyperplasia and family history [4]. The inherited predisposition to this malignancy has also been thoroughly studied to reveal two major susceptibility genes, BRCA1 and BRCA2 [5,6], as well as other gene mutations of lower penetrance [7-12]. However, these account for less than 5% of breast cancer cases, suggesting a more widespread relevance of common genetic variants in the population when considered cumulatively [13-17]. In addition, migrant and twin studies have hinted of an environmental component that can possibly overwrite the genetic influences on breast cancer, suggesting a multi-factorial nature of breast cancer risk or gene-environment interactions [18,19].

In an attempt to increase the clinical utility of these findings [20], statistical models have been designed and validated to aid in personalized risk assessment. Notably, the Gail model is the most widely used for breast cancer risk prediction. However, the model does not consider genetic factors directly [1] and has limited discriminatory power [21]. On the contrary, other studies in general have until recently ignored hormonal and reproductive characteristics of individual women [22,23]. Unfortunately, most of the models are still lacking in their predictive ability [24-27] and may be inapplicable to Chinese populations.

Despite having a much lower breast cancer incidence in Asia than in Europe [28], a rapidly increasing trend toward rates in the West is a cause for concern [29]. Singapore women are reported to have one of the highest rates of breast cancer incidence in the region with an annual increase of more than 3% [30]. As the linkage disequilibrium patterns differ among ethnic groups [31], findings from genome-wide association studies (GWAS) done on Caucasian populations [7,32-36] are potentially less relevant to Asian women [31,37-39]. In this nested case-control study, we incorporated a set of established GWAS risk alleles into a model with well-known lifestyle factors and evaluated its impact on predicting breast cancer risk in a Singapore Chinese cohort.

## Methods

### Study subjects

The subjects included in this study are women enrolled in the Singapore Chinese Health Study (SCHS), a population-based cohort study which has been described in detail previously [40]. Briefly, the cohort comprises 63,257 Chinese men and women between the ages of 45 to 74 who were recruited from 1993 to 1998. Participants were Singapore citizens or permanent residents who lived in government-built housing estates, and belonged to either of two major dialect groups: Hokkien or Cantonese. All participants were interviewed at baseline in their homes where they provided information on demographics, diet, level of physical activity, occupational exposure, smoking, and medical history. The women were also asked about their menstrual and reproductive history.

Between April 1994 and December 1999, blood and single-void urine specimens were collected from a random 3% sample of study enrollees. Details of the biospecimen collection, processing and storage procedures have been described previously [41]. Between January 2000 and April 2005, we extended our biospecimen collection to all surviving cohort members and collected biospecimens from 32,575 participants, representing a consent rate of about 60% of surviving cohort participants at that time.

Informed consent was obtained from all participants at baseline interview, as well as at time of biospecimen collection. The Institutional Review Board at the National University of Singapore has approved this study.

### Case ascertainment

Incident breast cancer cases were identified through the population-based cancer registry in Singapore. As of 28 June 2010, 941 had developed breast cancer in this cohort and among them, 414 donated blood previously and were included in this study. Compared with breast cancer patients who did not donate a blood sample, those who donated were younger at diagnosis (54.9 versus 56.0 years). Patients who did not donate blood samples were less educated (39.1% had no formal education) than those who did (25.1% had no formal education). There was also a higher proportion of family history of breast cancer among those who donated blood (n = 11, 2.66%) compared to those who did not donate (n = 4, 0.76%).

### Control selection

For each of the 414 breast cancer cases, up to three control subjects were randomly selected among all female cohort participants who had donated blood samples, and who were alive and free of breast cancer history at the time of cancer diagnosis of their index case. The chosen controls were matched to the index case on age at study enrollment (±3 years), dialect group (Hokkien, Cantonese), menopausal status at sample collection, dates of study enrollment (±2 years) and of blood collection (±6 months). For the 414 cases, there were six cases where only two eligible controls were found for each of them, and 408 cases where three controls were found for each of them.

### SNP selection, genotyping and quality control

We reviewed all published GWAS results related to breast cancer [42]. Single nucleotide polymorphisms (SNPs) from various studies [43-45], including more than 40 novel SNPs that were very recently identified from the Breast Cancer Association Consortium (BCAC) [46] and subsequently evaluated in a collaborative study on East Asian women [39], were evaluated for their application in breast cancer risk assessment. Due to differences in haplotype structure between Caucasian and Chinese populations, among SNPs in the same loci and having linkage disequilibrium (LD, r^2^) of more than 0.8 in HapMap Han Chinese in Beijing (CHB) population [47], the SNP with the greatest statistically significant association with breast cancer was genotyped, to ensure that all SNPs analyzed for risk prediction were independent of each other. SNPs with minor allele frequencies (MAF) less than 5% according to the Singapore Genome Variation Project (SGVP) [48] were also excluded.

Genotyping was done using the Sequenom iPLEX Gold MassARRAY system in 96-well plates (Sequenom, San Diego, CA, USA). MassARRAY Assay Design software was used to design amplification and extension primers (Sequenom). Multiplex PCR amplification was performed using Qiagen HotStart Taq DNA polymerase with 10 ng of genomic DNA (Qiagen, Germantown, MD, USA). Finally, primer extension reactions were carried out according to manufacturer’s guidelines. The investigators were blinded to the case/control status of the samples.

Of the initial 69 SNPs, seven SNPs (rs3803662, rs4808801, rs8100241, rs11199914, rs11814448, rs10069690 and rs1292011) could not be analyzed further as they produced poor, indistinguishable clusters, which could result in unreliable genotype callings. The average call rate for all SNPs was 98%, however the minor allele frequencies of rs11571833, rs132390, rs1045485, rs614367, rs999737 and rs8170 fell below the 1% threshold and were removed from analysis due to low power to detect any association with breast cancer. Deviation from Hardy-Weinberg equilibrium (*P* <0.0007) in controls was exhibited in the genotype distribution of rs7716600 and these SNPs were also discarded. Among the samples, three cases and fifteen controls did not meet the minimum call rate of 90%. The entire matched set was removed from analyses in the former. Therefore, 55 GWAS SNPs and 1,623 subjects (411 cases and 1,212 controls) were used for further data analyses.

### Data analysis

The SCHS questionnaire contained demographic data, reproductive risk factors, as well as information on diet and lifestyle. Risk factors to be included in the prediction model were selected according to results reported from other studies done on the SCHS cohort and factors used in the original Gail model [1]. Variables in the model were: level of education (no formal schooling, primary school, or secondary school or above), age at first live birth (<20, 20 to 24, 25 to 29 or nulliparous, ≥30 years), age at menarche (≥14, 12 to 13, <12 years), history of past breast biopsy (yes, no), family history (yes, no), body mass index (BMI) (<20, 20 to 23.9, 24 to 27.9, ≥28 kg/m^2^) and genetic risk score (GRS) in quartiles based on the controls. BMI was calculated as the weight divided by the squared height (kg/m^2^). Family history was limited to first-degree relatives only. The history of past breast biopsy (yes/no) was known for 218 (13.2%) women in the current nested case-control sample. For the remaining women with unknown history of breast biopsy, we imputed the value of history of breast biopsy variable by generating five values and picking the most frequent one, with BMI, estrogen and family history as predictors.

The association between breast cancer and demographic, reproductive, and other baseline characteristics was investigated using the Student’s *t* test and Mantel-Haenszel chi-squared test (linear by linear association) for continuous and categorical variables respectively. Established risk factors namely, parity, age at first live birth, age at menarche, age at menopause, BMI, family history, history of past breast biopsy and estrogen use were examined for their independent associations with breast cancer risk. A GRS was derived for each individual to represent the cumulative effect of the genetic variants on a woman’s risk of breast cancer. The Cochran’s Q test [49], which is the weighted sum of the squared difference between individual and pooled effects across studies, was used to test for heterogeneity among the current and published studies. The *P* values were obtained by comparing the statistic with a chi-square distribution with k-1 degrees of freedom, where k is the number of studies. SNPs were included in the computation of GRS only if heterogeneity was not statistically significant. If not, they were removed on the basis of inconsistency among studies (rs11780156, rs6504950, rs6001930, and rs2981579). To account for multiple hypothesis testing, a false discovery rate (FDR) correction according to the Benjamini-Hochberg procedure [50] was applied. A total of 51 SNPs was included in the computation of GRS. All SNP selection criteria had been decided *a priori*.

A fixed-effects meta-analysis of published GWAS and our study’s findings was performed in order to obtain reliable estimates for each SNP’s effect size in the form of a pooled odds ratio (pOR) derived from published and local studies. We weighted the effect size estimates of each study using the inverse of the corresponding standard errors of the respective studies. The GRS for an individual woman is equivalent to the sum of (log pOR of SNP) × (number of risk alleles that the individual carries for SNP) across all 51 SNPs. The GRS was normalized by dividing it by the average effect size of all SNPs in the population, as outlined previously [51]. Box plots were used to investigate the correlation between GRS and various breast cancer risk factors: age at first live birth, age at menarche, family history, past breast biopsy, BMI and education. Conditional logistic regression was used to calculate the crude and adjusted ORs with a 95% confidence interval for each risk factor. The *P* value for trend across categories was reported.

Variables in the Gail model and BMI were used to construct the conditional logistic regression model. The models with and without a GRS were compared in terms of their ability to accurately assess a woman’s five-year absolute risk. The probability that an individual *i* would be free of breast cancer beyond a certain time point, P_i_(t), was calculated as 1-(S_t_)^Ci^, where S_t_ is the proportion of people who were not diagnosed with breast cancer (survived) up to time point t. We estimated S_t_ using a Kaplan-Meier survival curve, based on data from the SCHS cohort - the cohort that this nested case-control study is from. Since a five-year risk is required in this study, *t* = 5 years. The individual-level coefficient, C_i_ is determined by the formula exp[∑β_j_(x_ij_ - μ_j_)], where β_j_ is the log odds ratio (OR) of the risk conferred by a variable j, and x_ij_ refers to the value of variable j for individual. The average for the variable in the population, μ_j_, was approximated using the average among controls. The benefit of adding genetic markers into the predictive model was assessed using a net reclassification improvement (NRI) index [52] that compares the risk classifications under models with and without GRS, to adjust the NRI index for overfitting, the index was further corrected using a bootstrap procedure [53].

As there is general expectation of a more reliable risk prediction model as additional risk variants become identified [27,54-56], we tested this hypothesis by rebuilding the model with six, nine, eleven, sixteen and fifty-one (this study) SNPs. These SNPs were chosen in an order in which their association with breast cancer risk was established through time [26,27,57-60].

Conditional logistic regression for the association between SNPs and breast cancer risk, NRI calculation and histogram plots were performed using R version 2.13.0. All other statistical analyses were performed using IBM SPSS version 21.0 (IBM Corp., Armonk, NY, USA). Statistical tests were two-sided and *P* <0.05 was considered statistically significant. In the test for heterogeneity, *P* <0.007 was used after accounting for multiple testing through Bonferroni correction.

## Results

**Figure 1:**
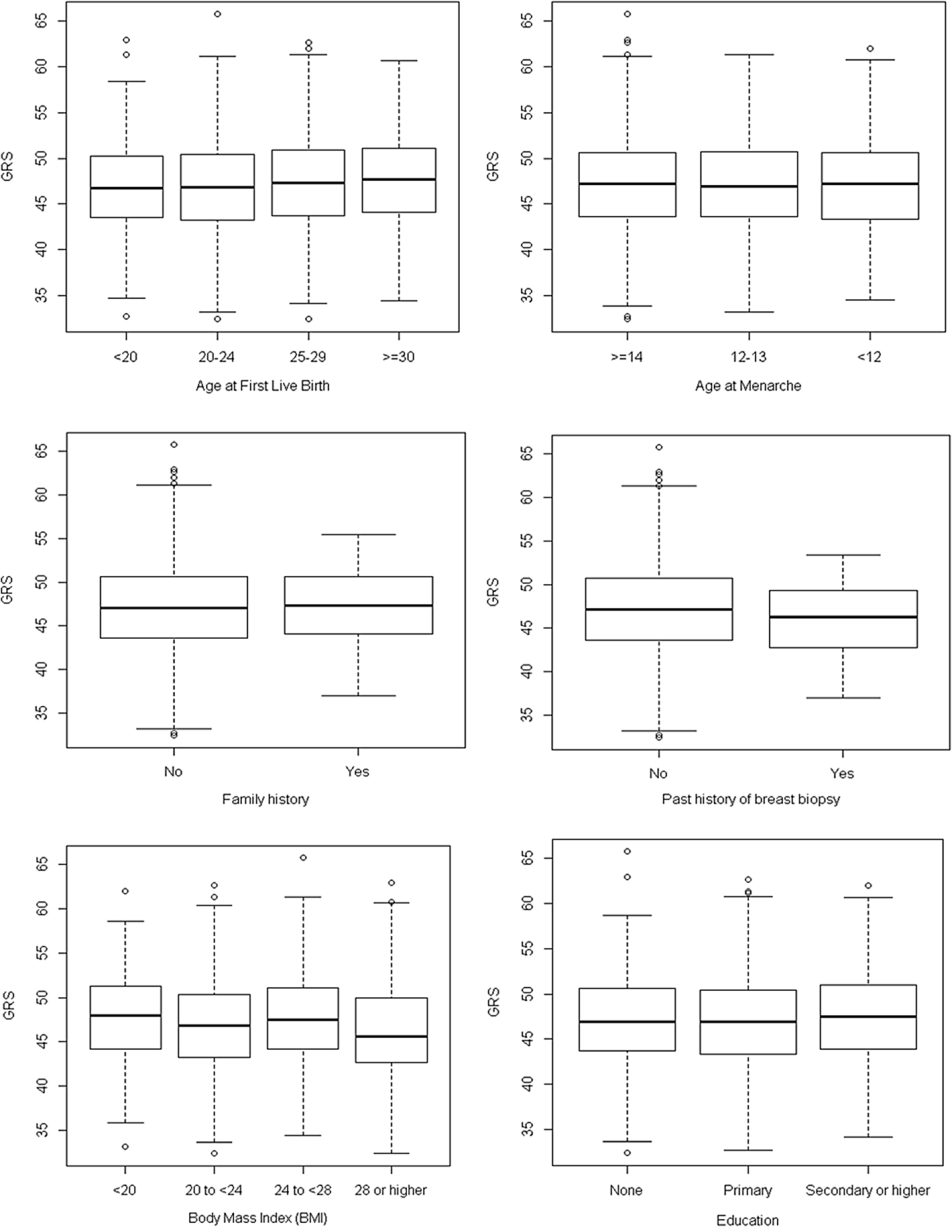
**Correlation ofgenetic risk score (GRS) with various breast cancer risk factors.** No statistically significant correlation was observed between GRS and the following breast cancer risk factors: age at first live birth, age at menarche, family history, past history of breast biopsy, body mass index (BMI) and education.

In total, 411 cases and 1,212 controls were used in the analyses. The distribution of subjects by background characteristics is shown in Table 1. As the cases and controls were matched on age and menopausal status, they were comparable in these aspects. Cases tend to be more well-educated (*P* = 0.003), older at first live birth (*P* = 0.022), report a younger age at menarche (*P* = 0.033), tend to be current estrogen users (*P* = 0.042) and fall under a higher GRS quartile (*P* < 0.001) compared to controls. They also have higher BMI (*P* = 0.066) and a positive family history of breast cancer (*P* = 0.063). The remaining factors comprising sleep and dietary patterns did not differ significantly between the two groups. Figure 1 displays no statistically significant correlation between GRS and the various breast cancer risk factors.

**Table 1:** Distribution of demographic and established risk factors for breast cancer in cancer patients (cases) and control subjects, The Singapore Chinese Health Study, 1993 to 1998

	**Cases (n = 411)**	**Controls (n = 1,212)**	P^*^
**Demographic factors**			
Mean age, years (SD)	54.9 (7.6)	54.9 (7.5)	0.964
Education level, n (%)			0.003
None	104 (25.3)	399 (32.9)	
Primary	186 (45.3)	518 (42.7)	
Secondary or higher	121 (29.4)	295 (24.3)	
**Reproductive risk factors**			
Number of live births, n (%)			<0.001
Nulliparous	56 (13.6)	85 (7.0)	
1-2	146 (35.5)	369 (30.4)	
3-4	131 (31.9)	483 (39.9)	
5 or more	78 (19.0)	275 (22.7)	
Mean age at first live birth, years (SD)	25.6 (4.9)	25.0 (4.7)	0.022
Mean age at menarche, years (SD)	14.1 (1.7)	14.3 (1.8)	0.033
Mean age at menopause, years (SD)	49.7 (4.4)	49.2 (4.3)	0.141
Estrogen use, n (%)			0.042
Non-user	372 (90.5)	1,131 (93.3)	
Ex-user	8 (1.9)	22 (1.8)	
Current user	31 (7.5)	59 (4.9)	
**Other risk factors**			
Family history of breast cancer, n (%)			0.063
No	400 (97.3)	1,196 (98.7)	
Yes	11 (2.7)	16 (1.3)	
History of past biopsy, n (%)			0.252
No	401 (97.6)	1,193 (98.4)	
Yes	10 (2.4)	19 (1.6)	
Mean body mass index, kg/m^2^ (SD)	23.5 (3.4)	23.2 (3.2)	0.066
Marine n-3 omega fatty acids, g (SD)	0.90 (0.4)	0.90 (0.5)	0.299
Vegetable-fruit-soy intake, n (%)			0.969
0	79 (19.2)	241 (19.9)	
1	99 (24.1)	307 (25.3)	
2	130 (31.6)	332 (27.4)	
3	103 (25.1)	332 (27.4)	
Green tea intake in tertiles, n (%)			0.418
None	234 (56.9)	713 (58.8)	
First	77 (18.7)	219 (18.1)	
Second	43 (10.5)	134 (11.1)	
Third	57 (13.9)	146 (12.0)	
Mean isoflavanoids, mg (SD)	19.4 (20.4)	19.8 (17.3)	0.700
Sleep duration, hours per day (SD)	7.0 (1.1)	7.0 (1.1)	0.685
Genetic risk score in quartiles, n (%)			
First (32.4 - 43.6)	80 (19.5)	326 (26.9)	<0.001
Second (43.6 - 47.1)	96 (23.4)	310 (25.6)	
Third (47.1 - 50.6)	109 (26.5)	296 (24.4)	
Fourth (50.6 - 65.8)	126 (30.7)	280 (23.1)	

*Two-sided *P* values were derived from chi-squared test for categorical variables and *t* test for continuous variables; the Mantel-Haenszel chi-squared test for trend with one degree of freedom for categorical variables with more than two levels. SD, standard deviation.

The ORs of the 51 SNPs from the current study and their pooled estimates with previous GWAS studies are displayed in Table 2. The SNPs that tag the *ESR1* loci - rs2046210 and rs3757318, presented ORs that were statistically significant at the 5% level. Associations with FOXQ1 (*P* = 0.088) and TOX3 (*P* = 0.098) displayed marginal significance. Apart from two tag SNPs for *ESR1*, another six SNPs (rs11552449, rs13387042, rs10759243, rs3903072, rs12422552, rs2236007) were also significantly associated with breast cancer in our study. Table S1 in Additional file 1 shows the corresponding pooled estimates when only published studies were used.

**Table 2:** The rare allele frequencies of 51 single nucleotide polymorphisms (SNPs) in breast cancer patients and control subjects and their association with risk of breast cancer, The Singapore Chinese Health Study, 1993 to 1998

**Chr**	**Genes in or near region**	**SNP**	**Effect allele**	**RAF in controls**	**RAF in cases**	**Per-allele OR (95% CI)**	**SE**	***P*** value^*^	**Pooled OR**	**FDR-corrected** ***P***_**het**_
1	PEX14	rs616488	G	0.348	0.361	1.06 (0.89, 1.26)	0.09	0.510	0.94	0.790
1	PTPN22/BCL2L15/AP4B1/DCLRE1B/HIPK1	rs11552449	A	0.596	0.556	0.85 (0.72, 0.99)	0.08	0.040	1.06	0.068
1	FCGR1B	rs11249433	G	0.029	0.026	0.85 (0.52, 1.39)	0.25	0.517	1.09	0.371
2	INHBB-	rs4849887	A	0.259	0.269	1.05 (0.87, 1.25)	0.09	0.634	0.92	0.561
2	METAP1D/DLX1/DLX2	rs2016394	A	0.195	0.191	0.96 (0.79, 1.18)	0.10	0.727	0.95	0.790
2	TNP1^‡^	rs13387042	G	0.907	0.883	0.77 (0.60, 0.99)	0.13	0.045	0.93	0.062
2	DIRC3	rs16857609	C	0.376	0.367	1.07 (0.90, 1.27)	0.09	0.462	1.08	0.977
3	ITPR1/EGOT	rs6762644	G	0.098	0.096	0.97 (0.73, 1.29)	0.14	0.841	1.07	0.790
3	SLC4A7	rs4973768	A	0.190	0.186	0.97 (0.80, 1.18)	0.10	0.757	1.11	0.473
3	TGFBR2	rs12493607	G	0.278	0.262	0.91 (0.76, 1.09)	0.09	0.290	1.06	0.619
4	TET2	rs9790517	A	0.613	0.635	1.09 (0.93, 1.29)	0.08	0.293	1.05	0.440
4	ADAM29	rs6828523	A	0.275	0.271	0.99 (0.82, 1.18)	0.09	0.879	0.90	0.729
5	5p12 (intergenic)	rs4415084	C	0.441	0.434	0.96 (0.82, 1.14)	0.08	0.668	1.15	0.087
5	MRPS30	rs10941679	G	0.499	0.519	1.09 (0.93, 1.29)	0.08	0.289	1.12	0.409
5	MAP3K1^‡^	rs889312	C	0.589	0.577	0.96 (0.82, 1.14)	0.08	0.666	1.05	0.727
5	RAB3C	rs10472076	G	0.247	0.260	1.07 (0.89, 1.28)	0.09	0.465	1.04	0.500
5	EBF1	rs1432679	G	0.631	0.642	1.05 (0.89, 1.24)	0.08	0.558	1.07	0.931
6	FOXQ1^†^	rs11242675	A	0.597	0.633	1.15 (0.98, 1.35)	0.08	0.088	-	-
6	ECHDC1/RNF146	rs2180341	G	0.181	0.198	1.09 (0.90, 1.33)	0.10	0.365	1.31	0.100
6	RANBP9	rs204247	G	0.588	0.611	1.10 (0.94, 1.29)	0.08	0.248	1.05	0.911
6	FAM46A	rs17529111	G	0.218	0.206	0.92 (0.75, 1.12)	0.10	0.404	1.06	0.675
6	ESR1	rs3757318	A	0.276	0.316	1.20 (1.01, 1.42)	0.09	0.039	1.04	0.790
6	ESR1^‡^	rs2046210	A	0.380	0.434	1.25 (1.06, 1.46)	0.08	0.008	1.27	0.970
7	ARHGEF5	rs720475	A	0.046	0.041	0.90 (0.61, 1.33)	0.20	0.596	0.94	0.941
8	RPL17P33	rs9693444	A	0.282	0.288	1.04 (0.87, 1.24)	0.09	0.691	1.07	0.977
8	8q24	rs13281615	G	0.502	0.500	0.99 (0.84, 1.16)	0.08	0.872	1.07	0.215
8	8q24	rs1562430	C	0.181	0.184	1.03 (0.83, 1.27)	0.11	0.820	0.87	0.273
9	CDKN2A	rs1011970	A	0.096	0.095	0.98 (0.75, 1.28)	0.14	0.875	1.07	0.639
9	KLF4	rs10759243	A	0.431	0.476	1.20 (1.02, 1.41)	0.08	0.027	1.06	0.790
9	KLF4	rs865686	C	0.057	0.067	1.19 (0.87, 1.64)	0.16	0.275	0.90	0.150
10	ZNF365	rs10822013	T	0.500	0.517	1.07 (0.91, 1.25)	0.08	0.409	1.08	0.387
10	ZNF365^‡^	rs10995190	A	0.012	0.013	1.14 (0.57, 2.30)	0.36	0.713	1.06	0.350
10	ZMIZ1	rs704010	A	0.369	0.397	1.12 (0.95, 1.33)	0.08	0.169	1.08	0.473
10	FGFR2^‡^	rs1219648	G	0.404	0.427	1.08 (0.92, 1.27)	0.08	0.343	1.14	0.560
10	FGFR2	rs2981582	A	0.338	0.370	1.14 (0.96, 1.34)	0.08	0.128	1.26	0.451
11	LSP1	rs3817198	G	0.095	0.108	1.14 (0.89, 1.46)	0.13	0.311	1.07	0.931
11	DKFZp761E198/OVOL1/SNX32/CFL1	rs3903072	A	0.210	0.175	0.81 (0.66, 0.99)	0.10	0.039	0.95	0.268
11	BARX2	rs11820646	A	0.489	0.509	1.09 (0.93, 1.28)	0.08	0.278	0.95	0.574
12	ATF7IP	rs12422552	C	0.273	0.325	1.29 (1.08, 1.53)	0.09	0.004	1.06	0.128
12	PTHLH	rs10771399	G	0.192	0.173	0.88 (0.71, 1.08)	0.11	0.221	0.86	0.729
12	NTN4	rs17356907	G	0.253	0.247	0.98 (0.82, 1.18)	0.09	0.839	0.91	0.604
14	PAX9/SLC25A21	rs2236007	A	0.297	0.255	0.83 (0.69, 0.98)	0.09	0.031	0.92	0.469
14	CCDC88C	rs941764	G	0.126	0.140	1.13 (0.90, 1.42)	0.12	0.308	1.06	0.931
16	TOX3	rs4784227	T	0.245	0.274	1.17 (0.97, 1.41)	0.09	0.098	1.23	0.574
16	TOX3	rs3112612	G	0.228	0.210	0.90 (0.74, 1.10)	0.10	0.300	1.13	0.066
16	CDYL2	rs13329835	G	0.053	0.054	1.02 (0.70, 1.46)	0.18	0.938	1.09	0.351
16	MIR1972-2/FTO	rs17817449	C	0.140	0.118	0.83 (0.66, 1.06)	0.12	0.134	0.93	0.079
18	AQP4^‡^	rs527616	G	0.267	0.266	0.99 (0.83, 1.19)	0.09	0.951	0.98	0.805
18	CHST9	rs1436904	C	0.486	0.509	1.09 (0.93, 1.28)	0.08	0.287	0.96	0.225
19	C19orf61:KCNN4:LYPD5:ZNF283	rs3760982	A	0.136	0.148	1.10 (0.87, 1.38)	0.12	0.437	1.06	0.994
21	NRIP1	rs2823093	A	0.035	0.032	0.90 (0.57, 1.40)	0.23	0.631	0.92	0.994

^†^A pooled odds ratio (pOR) was not obtained for rs11242675 due to significant heterogeneity with other published studies; ^‡^a pOR with the Asian studies was obtained due to heterogeneity with the results of European studies.^*^P value was derived from conditional logistic regression analysis. Chr, chromosome; RAF, risk allele frequency; FDR, false discovery rate.

The associations between breast cancer risk, established risk factors and GRS were evaluated. Compared to the lowest quartile, women in the highest GRS quartile were close to 80% (OR = 1.75, 95% confidence interval (CI) = 1.27 to 2.41) more likely to have breast cancer (Table 3). The magnitude of the association with GRS and the dose-response relationship remained almost unchanged even after adjusting for the established risk factors and education. Age at first live birth and age at menarche presented statistically significant trends with breast cancer risk, but were no longer significant after adjustment. On the contrary, the association of BMI with risk became significant after other factors were considered. After accounting for GRS and the above-mentioned risk factors, neither a positive family history nor a previous breast biopsy was significantly associated with breast cancer risk.

**Table 3:** The relation for genetic risk score and established conventional risk factors with risk of breast cancer, The Singapore Chinese Health Study, 1993 to 1998

**Variable**	**Case (n = 411), n (%)**	**Control (n = 1,212), n (%)**	**OR (95% CI)**	***P*** **value**	**Adjusted OR (95% CI)**^**ᶲ**^	***P*** **value**	**Adjusted OR (95% CI)**^**‡**^	***P*** **value**
Genetic risk score, quartiles (range), mean								
First (32.4-43.6), 40.4	80 (19.5)	326 (26.9)	1.00 (ref)		1.00 (ref)		1.00 (ref)	
Second (43.6-47.1), 45.4	96 (23.4)	310 (25.6)	1.26 (0.90, 1.75)	0.178	1.25 (0.90, 1.75)	0.182	1.26 (0.90, 1.76)	0.174
Third (47.1-50.6), 48.9	109 (26.5)	296 (24.4)	1,47 (1.06, 2.04)	0.020	1.49 (1.07, 2.06)	0.017	1.47 (1.06, 2.04)	0.022
Fourth (50.6-65.8), 53.6	126 (30.7)	280 (23.1)	1.78 (1.29, 2.44)	<0.001	1.74 (1.26, 2.40)	0.001	1.75 (1.27, 2.41)	0.001
*P* trend			1.21 (1.09, 1.33)	<0.001	1.20 (1.09, 1.33)	<0.001	1.20 (1.08, 1.33)	<0.001
Age at first live birth, years								
<20	56 (13.6)	212 (17.5)	1.00 (ref)		1.00 (ref)		1.00 (ref)	
20-24	129 (31.4)	458 (37.8)	1.11 (0.77, 1.59)	0.571	1.04 (0.72, 1.50)	0.823	1.05 (0.73, 1.51)	0.804
25-29 or null	175 (42.6)	416 (34.3)	1.67 (1.16, 2.39)	0.005	1.51 (1.04, 2.18)	0.030	1.49 (1.03, 2.15)	0.036
≥30	51 (12.4)	126 (10.4)	1.62 (1.03, 2.55)	0.038	1.46 (0.92, 2.31)	0.111	1.49 (1.03, 2.29)	0.131
*P* trend			1.25 (1.09, 1.42)	0.001	1.20 (1.05, 1.38)	0.008	1.19 (1.04, 1.37)	0.011
Age at menarche, years								
≥14	160 (38.9)	540 (44.6)	1.00 (ref)		1.00 (ref)		1.00 (ref)	
12-13	180 (43.8)	484 (39.9)	1.29 (1.00, 1.66)	0.049	1.21 (0.93, 1.56)	0.151	1.23 (0.94, 1.60)	0.126
<12	71 (17.3)	188 (15.5)	1.34 (0.95, 1.89)	0.097	1.20 (0.84, 1.71)	0.312	1.19 (0.83, 1.71)	0.345
*P* trend			1.18 (1.00, 1.39)	0.048	1.12 (0.94, 1.32)	0.208	1.11 (0.94, 1.33)	0.220
Family history								
No	400 (97.3)	1196 (98.7)	1.00 (ref)		1.00 (ref)		1.00 (ref)	
Yes	11 (2.7)	16 (1.3)	2.11 (0.97, 4.61)	0.061	2.06 (0.94, 4.52)	0.071	1.78 (0.79, 4.00)	0.162
Past breast biopsy, n (%)								
No	401 (97.6)	1193 (98.4)	1.00 (ref)		1.00 (ref)		1.00 (ref)	
Yes	10 (2.4)	19 (1.6)	1.55 (0.69, 3.48)	0.288	1.36 (0.60, 3.08)	0.462	1.36 (0.58, 3.20)	0.475
Body mass index, kg/m^2^								
<20	51 (12.4)	167 (13.8)	1.00 (ref)		1.00 (ref)		1.00 (ref)	
20 to <24	216 (52.6)	659 (54.4)	1.09 (0.77, 1.54)	0.632	1.11 (0.78, 1.58)	0.562	1.16 (0.81, 1.66)	0.423
24 to <28	102 (24.8)	305 (25.2)	1.10 (0.75, 1.62)	0.610	1.12 (0.76, 1.64)	0.558	1.15 (0.78, 1.70)	0.488
28 or higher	42 (10.2)	81 (6.7)	1.74 (1.07, 2.85)	0.027	1.83 (1.12, 3.01)	0.016	1.99 (1.21, 3.29)	0.007
P trend			1.14 (0.99, 1.31)	0.067	1.15 (1.00, 1.33)	0.048	1.17 (1.02, 1.35)	0.031

^ᶲ^Adjusted for education; ^‡^adjusted for education and all other factors in the table. OR, odds ratio; CI, confidence interval.

**Figure 2:**
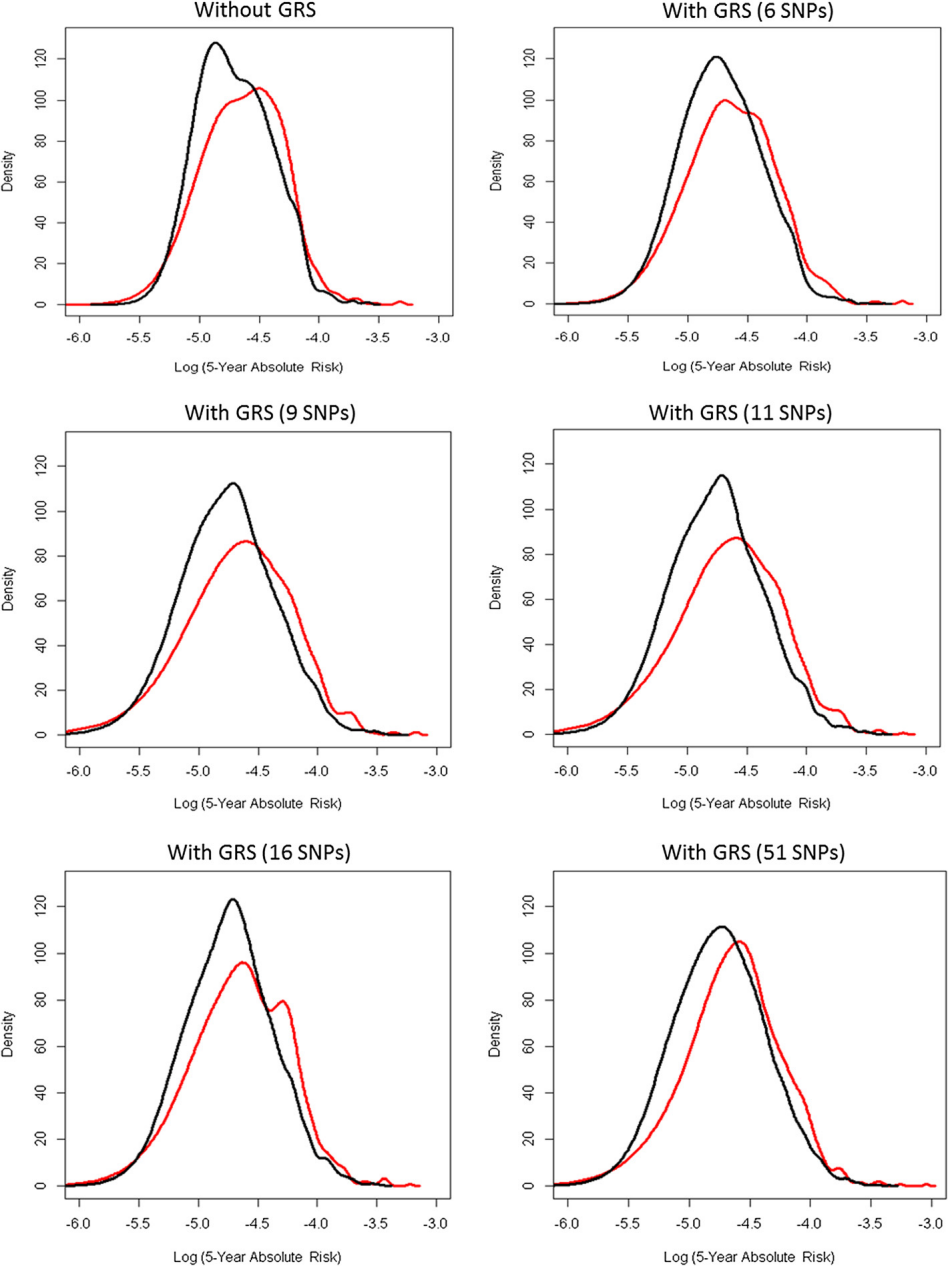
**Effect of increasing number of SNPs in breast cancer risk assessment.** The rate of increase in model discriminatory power (difference in log five-year absolute risk) between cases and controls diminishes as more SNPs are incorporated into the GRS. For instance, the change in improvement of model performance is minimal when the number of SNPs used in the GRS increased from 16 to 51. GRS is obtained by pooling the ORs of local and published studies. Y-axis is the density that reflects the frequency of subjects. (Black - controls, Red - cases). SNP, single nucleotide polymorphism; GRS, genetic risk score; OR, odds ratio.

A NRI index was used to assess the improvement in risk classification that would result from adding GRS to a model comprising established risk factors only. Table 4 shows the distribution of women across the various five-year absolute risk categories from <1.0% to ≥2.5%. Approximately 1,400 women reported five-year cumulative risks of less than 1.5%, while only 5.4% of cases and 2.0% of controls were assigned into high-risk groups of 2% or more. Among the 44 cases who were categorized as having a five-year absolute risk of 1.5% to <2.0% under the model without GRS, 12 of them were shifted to higher risk groups while 13 were moved to the lower risk category of 1.0% to <1.5% when GRS was added to the model. Similarly, for about 46.1% of the controls who were initially estimated to have 1.5% to <2.0% risk based on established risk factors only, the new model with GRS indicated a lower risk of 1.0% to <1.5%, while shifting 12 individuals to the 2.0% to <2.5% risk stratum. The reclassification improvement among cases was 11.2% (*P* <0.001), while that for controls was 2.2% (*P* = 0.04), though the latter was not statistically significant. This led to an NRI of 13.4% (*P* = 0.006), which decreased to 6.2% after correcting for optimism using a bootstrap method. This meant, that overall, 6% of women were reclassified into more appropriate risk groups when a genetic component was considered.Figure 2 demonstrates the change in model discriminatory power as GRS that represented the cumulative effect of six, nine, eleven, sixteen and fifty-one (this study) SNPs were used in predicting the five-year absolute risk. A marginal improvement in model performance was noted.

**Table 4:** Reclassification of five-year absolute risk of breast cancer based on a risk model containing Gail variables with and without genetic risk scores (GRS) on 411 breast cancer patients and 1,212 healthy women, The Singapore Chinese Health Study, 1993 to 1998

**Five-year risk without GRS (%)**	**Five-year risk with GRS (%)**					
	**<1.0**	**1.0 - <1.5**	**1.5 - <2.0**	**2.0 - <2.5**	**≥2.5**	**Total**
<1.0						
Control	511	84	0	0	0	595
Case	109	46	0	0	0	155
1.0 - <1.5						
Control	90	356	33	0	0	479
Case	36	123	39	0	0	198
1.5 - <2.0						
Control	0	53	50	12	0	115
Case	0	13	19	12	0	44
2.0 - <2.5						
Control	0	0	12	3	1	16
Case	0	0	4	2	3	9
>2.5						
Control	0	0	0	2	5	7
Case	0	0	0	1	4	5
Total						
Control	601	493	94	18	6	1,212
Case	145	182	62	15	7	411

## Discussion

We have evaluated a total of 51 SNPs and constructed a GRS to reflect their cumulative effect on breast cancer risk. The genetic score was independently associated with breast cancer risk after adjusting for education and other established risk factors. These common genetic markers, when considered in aggregate, together with reproductive factors and BMI, can improve the risk stratification for close to 10% of Singapore Chinese women. Similar to past SCHS studies [61,62], cases tend to be more highly educated compared to the controls, hence the adjustment for education in the conditional logistic regression model. However, BMI and family history did not differ significantly between the two groups although the direction of the associations was in agreement with prior knowledge. Failure to reach statistical significance is very likely due to the sample size, hence limiting our study’s power to detect an association. An attenuated effect of family history (OR = 1.78) after accounting for GRS was noted too. This could be due to the fact that risk variants, which are directly or indirectly incorporated into the GRS are also traits that tend to be inherited together.

To our knowledge, we have investigated the largest number of SNPs for use in risk assessment in an Asian population. Studies by Dai *et al.*, Sueta *et al.*, and Zheng *et al.* have reported the discriminatory power of using five, seven and eight SNPs respectively [37,54,55]. All groups demonstrated the clinical utility that can potentially be achieved with the incorporation of common genetic variants to a model containing established risk factors. Locally, a group has reported the potential effect of combining eight SNPs with clinicopathological factors in risk prediction for a Singapore Chinese population [56]. Likewise, we have shown that common genetic markers, when considered in aggregate, together with reproductive factors and BMI, can improve the risk stratification for close to 10% of Singapore Chinese women. However, the majority of the cases (79.6%) presented low five-year cumulative risks of less than 1.5% after GRS was considered, with only 5.4% being assigned five-year cumulative risks of 2% or more. This suggests that other genetic, physiological and environmental factors not accounted for in this study, which includes novel factors yet to be identified, still account for a large proportion of risk for breast cancer in this population.

To gauge how much value a genetic component can add to an individual’s risk assessment, we included the GRS into a model consisting of Gail variables and compared its performance with the model without GRS. Many of the studies published thus far have reported the accuracy of their models in terms of area-under-the-curve (AUC) values [63]. However, AUC is insensitive even when strong predictors are added to the model [64], hence could partially account for insignificant increases in model discrimination. It also does not provide information about the actual risks predicted, therefore, its direct clinical relevance is limited [65]. Instead, we have quantified the degree of correct risk reclassification by calculating the NRI index [52]. Even though NRI depends on arbitrary cutoff points, it is robust to moderate changes [66]. Bootstrapping was performed to account for overfitting of our data to the model, which could in turn lead to an overestimation of model performance. Although we attempted to incorporate all variables of the Gail model in our study, the low uptake of screening mammography meant that we did not have breast biopsy information for a majority of our participants. This problem of ‘missing data’ was overcome by imputing breast biopsy status based on BMI, estrogen use and family history - variables that differed between those who ever and never had a breast biopsy.

SNPs that tagged the *ESR1* gene, which codes for estrogen receptor alpha (ESRα), presented statistically significant associations and consistent ORs in our study. This is in concordance with findings from other groups, which showed substantial effect sizes for SNPs in this region, highlighting a likely association with breast cancer among Chinese and Japanese women [67]. The study conducted among Singaporean Chinese also reported that rs2046210 recorded the largest magnitude, similar to our current study [56]. This is in agreement with a comparison study by Hein *et al.*, which found significant association of the 6q25.1 locus in both Asians and Europeans but greater effects in the former [68]. Our study also supports the finding that the *MAP3K* SNP, rs889312, does not increase the risk of breast cancer among the Chinese [31,38,56], contrary to that of European populations [7,33]. However, unlike the other studies [7,8,31,33,37,38], statistical significance was not observed here for another well-established susceptibility loci containing FGFR2.

The OR of rs11242675 (*FOXQ1*) reported here was 1.15. This is contradictory to the findings of many published works, which have reported statistically significant protective effects, but the risk effect we found is supported by the most recent BCAC study [46]. As a result, significant heterogeneity was observed among the various studies and a pOR was not applicable. Forkhead box Q1 (FOXQ1) is a transcription factor found on the 6p25 locus. Overexpression of the protein has been shown to enhance tumorigenicity and tumor growth through its angiogenic and anti-apoptotic properties [69]. Its novel role in the metastasis of breast cancer has also been suggested [70]. In view of a plausible biological function of FOXQ1 in promoting cancer aggression, as well as marginal statistical significance that was a likely consequence of small sample size, rs11242675 was included in the GRS for risk assessment.

Another SNP that was also considered in the GRS due to its marginally significant *P* value (*P* = 0.098) was rs4784227. Rs4784227 is situated at 16q12.1 [71] and has been predicted to interfere with the affinity of FOXA1, an essential component of ESRα signaling [72], to its binding site [73]. Its position in a regulatory region that interacts with the *TOX3* promoter enables it to disrupt the expression of this gene, which in turn alters chromatin structure and DNA-protein binding patterns essential for cell survival [71]. An OR of 1.17 was seen in our study. This effect size and direction were similar to the findings of others thus a pOR was used for GRS computation.It was observed, in Figure 2, that the marginal improvement in model performance was not proportional to the increase in additional SNPs used. Although the discovery of additional SNPs do not drastically improve the assessment of breast cancer risk, this is expected since the first few new SNPs discovered would have been associated with much larger effect sizes. Also, as the cost of genotyping continues to decrease, we expect the use of additional SNPs in risk assessment to be cost-effective in the near future.

This study has several strengths. The study was nested within a population-based prospective cohort that provides the use of questionnaire data collected before the occurrence of breast cancer to reduce recall and reverse causality bias. The inclusion of genetic variants in risk assessment is advantageous as it is not subjected to time-dependent errors in measurement, unlike environmental exposures such as BMI or smoking. We have also shown the strength of the association between GRS and breast cancer risk; it remains virtually unchanged even after all other established risk factors have been considered, highlighting the importance of genetics in this aspect.

There are also several limitations in our study. The small sample size of approximately 1,600 women has made it difficult to attain statistical significance for most of the SNPs that were identified in GWAS studies. Nevertheless, the direction of the effects of most SNPs was consistent with the published literature. As all the subjects recruited were Chinese women, this could restrict the generalizability of our results. Studies will need to be conducted in larger populations and among women of other ethnicities to validate the effect of these polymorphisms. We were not able to consider two factors in this study: 1) the presence of copy number variations (CNVs) and their potential effects on breast cancer risk and 2) the various subtypes of the disease. Given the proximity of some SNPs to CNV regions [74], and the relation between CNVs and familial breast cancer [75], an effect of CNVs on risk of disease is not unlikely. However, modeling this poses difficulties and may not alter the results substantially [74]. Although further analysis by disease subtype would have been ideal, we were restricted by the study’s limited sample size and power. Finally, the breast cancer cases included in this study from the cohort had a higher prevalence for positive family history of breast cancer compared to cases that were not included in this study, although this prevalence was still generally low (2.7%).

## Conclusions

In summary, we have shown the extent to which 51 SNPs may improve the current assessment of breast cancer risk. Most of the SNPs identified in other Western and Asian studies have presented similar effect sizes in our Singapore Chinese population. Despite conferring minimal increase in risk, individual genetic variants when considered cumulatively can result in considerable effects, leading to improved risk stratification. By including a genetic component for risk assessment, more targeted measures of prevention and screening can be implemented. For countries such as Singapore where breast cancer incidence is relatively low and mammography screening is not as well-received, cost-efficiency and ethical issues can be more aptly addressed.

## Abbreviations

AUC: area under the receiver operating characteristic curve; BCAC: Breast Cancer Association Consortium; BMI: body mass index; CHB: Han Chinese in Beijing; CI: confidence interval; CNV: copy number variation; ESRα: estrogen receptor alpha; FDR: false discovery rate; GRS: genetic risk score; GWAS: genome-wide association studies; MAF: minor allele frequency; NRI: net reclassification improvement; OR: odds ratio; pOR: pooled odds ratio; RAF: risk allele frequency; SCHS: Singapore Chinese Health Study; SGVP: Singapore Genome Variation Project; SNP: single nucleotide polymorphism.

## Competing interests

The authors declare that they have no competing interests.

## Authors’ contributions

MH directed the study and was responsible for the study design. CPLL performed data management, statistical analyses, interpretation of results and drafted the initial manuscript. AI managed the genotype data and performed statistical analyses. AS was involved in the statistical analyses and interpretation of results. JL administrated the genotyping analysis. JY and WPK were responsible for biospecimen and data collection for the Singapore Chinese Health Study cohort, on which this study is based. All authors read, critically revised and approved the final manuscript.

## Supplementary Material

Additional file 1: Table S1 The corresponding individual and pooled ORs from published GWAS studies for 51 SNPs. This table presents the individual and pooled ORs, as well as FDR-corrected *P*-het of each SNP from published studies. The pooled ORs for each SNP were obtained from published studies only. Click here for file
